# Efficacy and safety of belimumab therapy for patients with lupus nephritis: A meta‐analysis and a propensity score‐matched case–control study

**DOI:** 10.1002/iid3.954

**Published:** 2023-07-27

**Authors:** Kai Zhang, Tiening Qi, Donghua Guo, Youxia Liu

**Affiliations:** ^1^ Department of Nephrology Tianjin Medical University General Hospital Tianjin People's Republic of China; ^2^ Department of Operating Room Tianjin Medical University General Hospital Tianjin People's Republic of China; ^3^ Department of Nephrology Tianjin Medical University General Hospital Airport Hospital Tianjin People's Republic of China

**Keywords:** a propensity score‐matched case–control study, belimumab, lupus nephritis, meta‐analysis, randomized controlled trials

## Abstract

**Objective:**

In this study, we performed a meta‐analysis and a propensity score‐matched case–control study to evaluate the efficacy and safety of belimumab in patients with lupus nephritis (LN).

**Methods:**

We analyzed the data from three randomized controlled trials (RCTs) to assess the effects of belimumab treatment on renal improvement and adverse effects. Our study included a total of six LN patients who received belimumab treatment and an additional six LN patients who received standard therapy. All participants were followed up for a duration exceeding 96 weeks to evaluate the outcomes of the treatments.

**Results:**

Our meta‐analysis incorporated data from three articles involving a total of 666 patients. The results of the analysis revealed that a higher proportion of patients who received belimumab treatment experienced renal improvement compared to those in the control group. The patients in belimumab group had a higher renal complete response rate and proteinuria improvement rate compared to the control group. However, belimumab treatment did not increase the renal partial response rate compared to the control group. The belimumab group also exhibited a higher proportion of patients who achieved normalization of antidouble‐stranded DNA, as well as normalization of low C3 and C4 levels. In our case–control study, significant improvement in proteinuria was demonstrated with belimumab at Week 48 (*p* = 0.037) and at all subsequent time points (all *p* < 0.05). Over the course of 96 weeks, belimumab treatment was associated with renal function stabilization and an increase in C3 and C4 levels. Moreover, the use of belimumab resulted in a reduction in glucocorticoid dosage at Week 24 (*p* = 0.02). Additionally, patients receiving belimumab treatment had a lower risk of severe treatment‐emergent adverse events, and no other significant adverse effects were observed between the two groups.

**Conclusions:**

In patients with LN, the utilization of belimumab therapy has demonstrated notable improvements in renal response rates. Additionally, it has shown a decreased likelihood of serious treatment‐related adverse events and a diminished need for glucocorticoid dosage when compared to the active control group.

## Graphical abstract

Belimumab increased the rates of renal response in patients with lupus nephritis. Belimumab decreased the risk of severe treatment‐emergent adverse events and the dose of glucocorticoid compared to active control in patients with lupus nephritis.

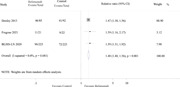

## 1 INTRODUCTION

Systemic lupus erythematosus (SLE) is indeed an autoimmune disease that can affect multiple organs and is more common in young women. It is characterized by the production of autoantibodies that target various self‐antigens in the body.^1^, ^2^, ^3^ Lupus nephritis (LN) is a common manifestation of SLE and has a significant impact on the long‐term prognosis of affected individuals.^4^, ^5^ If poorly controlled, LN can progress to end‐stage renal disease in around 10% of cases within 5 years of onset.^6^, ^7^ The management of LN has evolved over the years, leading to improved outcomes for patients. The introduction of corticosteroids and later immunosuppressive agents such as cyclophosphamide and other immunosuppressors has had a significant impact on the survival of patients with proliferative LN. These medications help to control inflammation, reduce immune system activity, and slow down the progression of kidney damage. Despite the standard use of these agents, a subset of patients may exhibit an incomplete response to therapy. This highlights the need for continued research and development of more effective therapies to improve outcomes in LN. To address these challenges, ongoing efforts are focused on refining and individualizing treatment approaches. The advancement of LN treatment is now focused on identifying therapies that can improve long‐term renal outcomes and reduce treatment‐related toxicity.^8^, ^9^, ^10^, ^11^

Medications targeting B cells have been investigated as potential treatments for SLE. Belimumab is a recombinant immunoglobulin G‐1 monoclonal antibody that specifically targets soluble B‐cell activating factors (BAFF), a cytokine that plays a critical role in promoting B‐cell survival and activation. It is approved for the treatment of SLE in patients with active disease.^12^, ^13^, ^14^ Clinical trials and observational studies have examined the efficacy and safety of belimumab in LN. Belimumab has shown promising results in improving kidney outcomes in patients with LN. Studies have demonstrated that the addition of belimumab to standard therapy leads to increased renal response rates and reduced risk of renal flare in patients with LN.^15^, ^16^, ^17^ Belimumab has received approval in the United States and Europe for the treatment of LN. The approval signifies that belimumab can be considered as an option in the management of LN, particularly in combination with standard therapy. While clinical trials provide valuable information on the efficacy of belimumab in the treatment of LN, the real‐world effectiveness of the medication may differ.

A meta‐analysis conducted on patients with LN demonstrated that the addition of belimumab to standard treatment was associated with a beneficial renal response in patients with LN and did not show an increased risk of adverse effects compared to standard treatment alone. However, it is important to note that this meta‐analysis only included two trials, and some relevant studies were not included. Further research incorporating a broader range of studies is needed to obtain a more comprehensive understanding of the effects of belimumab treatment in patients with LN.

Therefore, to provide a more comprehensive analysis, we conducted a literature review and assessed the efficacy and safety of belimumab in patients with LN based on randomized controlled trials (RCTs) as well as real‐world data specifically from China.

## 2 METHODS

### 2.1 Data sources, search strategy, and selection criteria

To identify relevant studies for our systematic review, a comprehensive literature search was conducted in three databases: MEDLINE (Ovid; from January 1950 to May 2022), EMBASE (from January 1970 to May 2022), and the Cochrane Library database (Cochrane Central Register of Controlled Trials [CENTRAL]; no date restriction). We used several keywords including “belimumab,” “lupus nephritis,” “randomized controlled trial,” “RCT,” “kidney outcome,” and “adverse events” in our search strategy. Furthermore, we reviewed the reference lists of the retrieved articles to identify any additional relevant research that might have been missed in the initial search. The inclusion criteria for the studies in our review were as follows: (1) patients diagnosed with LN; (2) RCTs conducted on the topic; (3) trials that compared outcomes between belimumab and other active controls; (4) trials that reported outcomes of interest, including kidney outcomes and adverse events. To maintain transparency and adhere to best practices, this study followed the PRISMA (Preferred Reporting Items for Systematic Reviews and Meta‐Analysis) guidelines.^18^

### 2.2 Quality assessment and data extraction

The literature review, data extraction, and quality assessment were conducted by two researchers independently, following a standardized methodology. Any discrepancies that arose during these steps were resolved through dialog with a third author to reach a consensus. The data extraction process focused on capturing relevant information related to the following aspects: (1) *patient characteristics*: age, gender, urine protein‐to‐creatinine ratio, proteinuria, estimated glomerular filtration rate (eGFR), serum creatinine, serum albumin, and SLE disease activity index score; (2) *study design*: study period and country, sample sizes, and duration of follow‐up; (3) *treatment details*: dose, route, and regimen; (4) efficacy and safety outcomes. The quality assessment of included RCTs was evaluated using the Cochrane Collaboration instrument for assessing bias.^19^

### 2.3 Outcomes

The outcomes assessed in the systematic review were classified according to the definition provided by each study. They include renal improvement, renal complete response, renal partial response, proteinuria improvement, antidouble‐stranded DNA (anti‐dsDNA) antibody (positive to negative), C3 normalization, C4 normalization, and adverse effects. Adverse effects comprised serious treatment‐emergent adverse events (TEAEs), infectious TEAEs, and death.

### 2.4 A propensity score‐matched case–control study

There were 48 patients diagnosed with LN by renal biopsy in the Department of Nephrology at Tianjin Medical University General Hospital from January 2019 to June 2020. A total of 12 patients received a combination of belimumab and standard therapy, of these four patients discontinued the treatment due to high treatment costs or were lost to follow‐up. Additionally, two patients did not complete the full 2‐year follow‐up period. As a result, the analysis and summary presented here focus on the remaining six patients who had complete information available for the entire 2‐year follow‐up. Patients received intravenous belimumab 10 mg/kg on Days 0 (baseline), 14, and 28, and every 28 days thereafter. The standard therapy consisted of oral glucocorticoids and mycophenolate mofetil and/or tacrolimus, which were used for both induction and maintenance phases of treatment.

We used stratified sampling to identify 6 matched patients who received standard therapy alone during the same time interval. To perform stratified sampling, patients were stratified by age, gender, proteinuria, and eGFR. Computer‐generated random numbers were then used to select patients from each of these strata.

The clinical information collected for the study encompassed various factors, such as age, gender, systemic pressure, proteinuria, serum creatinine, and eGFR. Additionally, laboratory biomarkers, LN kidney biopsy class, and treatment details were also included in the analysis.

The study was approved by the Ethics Committee of Tianjin Medical University General Hospital, and all participants provided written informed consent.

### 2.5 Statistical analysis

The differences between the two groups were compared using a *t* test or nonparametric test for measurement data, according to the distribution of the variables. Using the random‐effects model, the odds ratio (OR) and 95% confidence interval (CI) for each outcome were calculated. The *I* ^2^ metric was utilized to quantify the proportion of inconsistency. Egger test and Begg funnel plots of the natural log of the OR versus its standard error were utilized to evaluate the possibility of publication bias (SE). A two‐sided *p* value of less than 0.05 was considered statistically significant. All meta‐analyses were conducted utilizing STATA (version 13.0; Stata Corp.).

## 3 RESULTS

### 3.1 Study flow and characteristics of included studies

**Figure 1:**
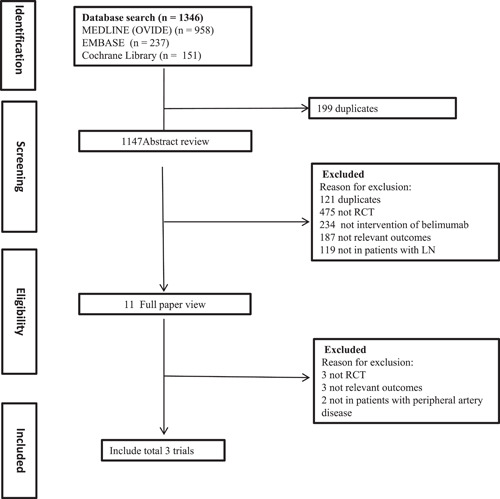
Process for identifying studies eligible for the meta‐analysis. LN, lupus nephritis; RCT, randomized controlled trial.

Our literature search yielded a total of 1346 relevant articles. After screening their titles and abstracts, 11 full‐text articles were further assessed for eligibility. Finally, three articles met the inclusion criteria and were included in the meta‐analysis (Figure 1). These three articles involved a total of 666 patients.^20^, ^21^, ^22^ The selected trials were published between 2013 and 2021, providing a recent overview of the efficacy and safety of belimumab in patients with LN. The duration of follow‐up varied among the trials, with a median range of 48–104 weeks. The average age of the patients included in the studies was 33 years. The treatment schedule varied slightly across the studies. In one study, patients were administered belimumab at Weeks 0 (baseline), 14, and 28, and then every 28 days thereafter until Week 72. In another study, patients received belimumab at Weeks 0 (baseline), 14, and 28, and then continued to receive it every 28 days until Week 100. Additionally, in one of the studies, the dosing regimen of belimumab was slightly modified. Patients received an initial dose of 10 mg/kg at Weeks 4, 6, and 8, and then subsequently received the medication every 4 weeks until Week 48. Table 1 provides comprehensive information on patient demographics and study design. The specific dosage and frequency of administration were outlined in Table 2 for each study.

**Table 1:** Characteristics of included studies.

References	Phase	Country	Control/belimumab									
			Mean age (years)	Female (%)	UPCR	Proteinuria (g/24 h)	eGFR (mL/min/1.73m^2^)	Serum creatinine (μmol/L)	Serum albumin (mg/dL)	SLEDAI score	Sample size	Follow‐up (weeks)
Dooley et al.^20^	Phase III	Multinational	NA	NA	1.8 ± 1.7/1.7 ± 1.2		NA	74.8 ± 23.9/74.7 ± 23.4	NA	12.9 ± 4.6/12.9 ± 4.3	92/85	52
BLISS‐LN^22^	Phase III	Multinational	33.1 ± 10.6/33.7 ± 10.7	87.8/88.3	3.5 ± 3.6/3.2 ± 2.7		101.0 ± 42.7/100.0 ± 37.7	NA	NA	12.2 ± 4.8/12.5 ± 5.3	223/223	104
Atisha‐Fregoso et al.^21^	Phase II	USA	32.3 ± 11.43/34.5 ± 9.14	81.8/90.5	3.4 ± 1.5/3.3 ± 2.5		92.7 ± 36.0/89.1 ± 33.9	90.2 ± 36.2/91.9 ± 41.4	2.96 ± 0.50/2.89 ± 0.61	NA	22/21	48

Abbreviations: BLISS‐LN, Belimumab International Study in Lupus Nephritis; eGFR, estimated glomerular filtration rate; NA, not available; SLEDAI, systemic lupus erythematosus disease activity index; UPCR, urine protein‐to‐creatinine ratio.

**Table 2:** Treatment of included studies.

References	Control/belimumab				
	Corticosteroid use (%)	Immunosuppressant use (%)	Belimumab regimen	Belimumab usage	Control regimen
Dooley et al.^20^	93.5/95.3	56.5/63.5	Belimumab plus standard therapy	At a dose of 10 mg/kg of body weight on Days 0 (baseline), 14, and 28, and every 28 days thereafter to Week 72	Standard therapy
BLISS‐LN^22^	NA	NA	Belimumab plus MMF or CYC	At a dose of 10 mg/kg of body weight on Days 1 (baseline), 15, and 29, and every 28 days thereafter to Week 100	Placebo plus MMF or CYC
Atisha‐Fregoso et al.^21^	NA	NA	Belimumab plus Rituximab and CYC	At a dose of 10 mg/kg at Weeks 4, 6, and 8, and every 4 weeks thereafter through Week 48.	Rituximab and CYC

Abbreviations: BLISS‐LN, Belimumab International Study in Lupus Nephritis; CYC, cyclophosphamide; MMF, mycophenolate mofetil; NA, not available.

### 3.2 Quality of studies

The quality assessment of each study was conducted using the Cochrane risk of bias tool, which evaluates various aspects of the study design to identify potential sources of bias. This tool assesses key domains such as sequence generation, allocation concealment, performance bias, detection bias, incomplete outcome data, and selective reporting, among others. Supporting Information: Figure 1 provides an overview of the risk of bias for the included studies.

### 3.3 Association of belimumab with renal improvement

**Figure 2:**
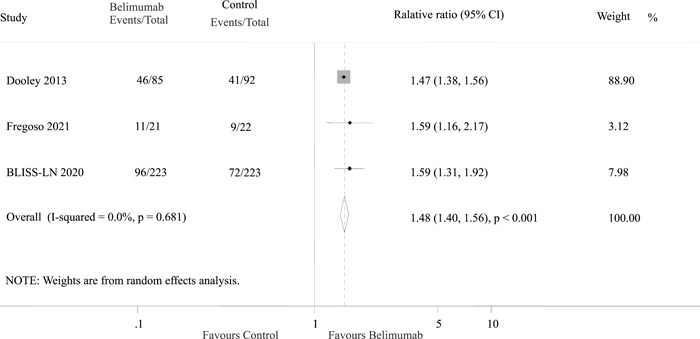
Effect of belimumab on renal improvement in patients with lupus nephritis. BLISS‐LN, Belimumab International Study in Lupus Nephritis; CI, confidence interval.

The outcome of renal improvement was reported in three studies, encompassing a total of 275 patients. Among these patients, 153 (46.5%) belonged to the belimumab group, while 122 (36.2%) were part of the control group. The results indicated that a higher proportion of patients who received belimumab experienced renal improvement compared to those in the control group (odds ratio [OR]: 1.48; 95% confidence interval [CI]: 1.40–1.56; *p* < 0.001) with no evidence of heterogeneity among the study results (*I* ^2^ = 0.0%, *p* = 0.68; Figure 2).

### 3.4 Association of belimumab with renal complete response and partial response

**Figure 3:**
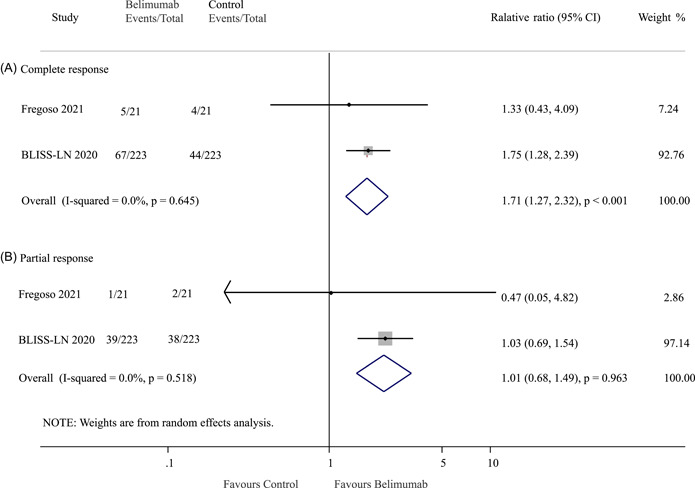
Effect of belimumab on (A) complete response and (B) partial response in patients with lupus nephritis. BLISS‐LN, Belimumab International Study in Lupus Nephritis; CI, confidence interval.

Two trials included data on the outcomes of complete renal response and partial renal response. Among the patients receiving belimumab, 72 individuals (29.6%) achieved a renal complete response, while in the control group, 48 patients (19.7%) achieved the same outcome. The analysis showed that the belimumab group had a significantly higher rate of renal complete response compared to the control group (OR: 1.71; 95% CI: 1.27–2.32; *p* < 0.001) with no evidence of heterogeneity (*I* ^2^ = 0.0%, *p* = 0.645; Figure 3A).

Among the patients receiving belimumab, 40 individuals (16.4%) achieved a partial renal response, while in the control group, an equal number of 40 patients (16.4%) achieved the same outcome. The analysis did not show a significant difference between the two groups regarding the partial renal response rate (OR: 1.01; 95% CI: 0.68–1.49; *p* = 0.963; *I* ^2^ = 0.0%, *p* = 0.518; Figure 3B).

### 3.5 Association of belimumab with proteinuria improvement

**Figure 4:**
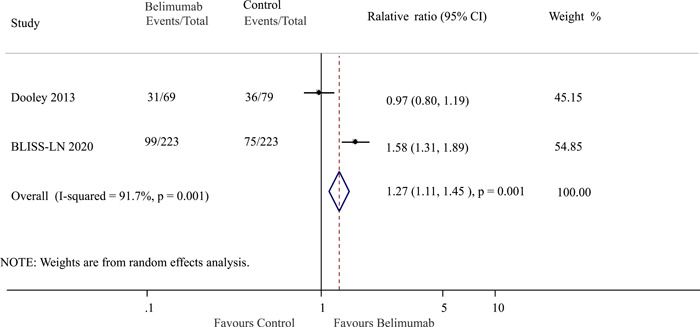
Effect of belimumab on proteinuria improvement in patients with lupus nephritis. BLISS‐LN, Belimumab International Study in Lupus Nephritis; CI, confidence interval.

In the two trials that reported data on proteinuria improvement, a total of 241 patients were included. Among these patients, 130 individuals (44.5%) belonged to the belimumab group, while 111 patients (36.8%) were part of the control group. The analysis revealed that a higher percentage of patients in the belimumab group experienced an improvement in proteinuria compared to the control group (OR: 1.27; 95% CI: 1.11–1.45; *p* = 0.001; *I* ^2^ = 91.7%, *p* = 0.001, Figure 4).

### 3.6 Association of belimumab with anti‐dsDNA antibody and C3 and C4 levels

**Figure 5:**
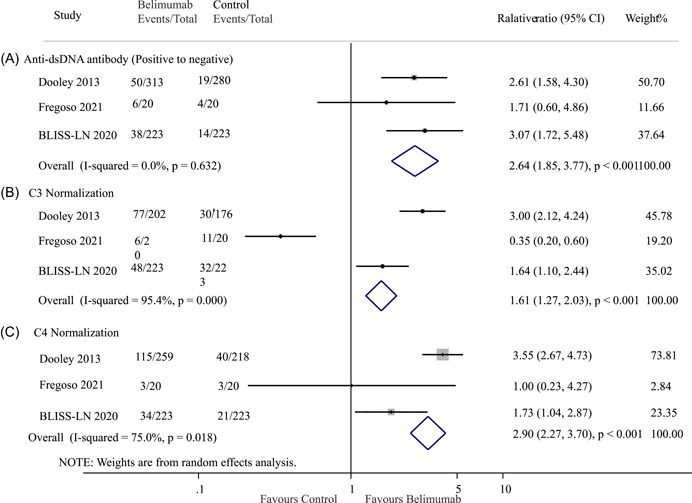
Effect of belimumab on (A) anti‐dsDNA antibody, (B) C3 and (C) C4 levels in patients with lupus nephritis. BLISS‐LN, Belimumab International Study in Lupus Nephritis; CI, confidence interval; dsDNA, double‐stranded DNA.

Regarding the positive‐to‐negative conversion rate for anti‐dsDNA antibodies, the combined results showed a significantly higher rate in the belimumab group compared to the control group (OR: 2.64; 95% CI: 1.85–3.77; *p* < 0.001; *I* ^2^ = 0.0%, *p* = 0.632; Figure 5A). Similarly, the analysis revealed a higher likelihood of achieving normalization of low C3 levels (OR: 1.61; 95% CI 1.27–2.03; *I* ^2^ = 95.4%, *p* = 0.000; Figure 5B) and low C4 levels (OR: 2.90; 95% CI: 2.27–3.70; *p* < 0.001; *I* ^2^ = 75.0%, *p* = 0.018; Figure 5C) in the belimumab group compared to the control group.

### 3.7 Association of belimumab with serious TEAEs, infectious TEAEs, and death

**Figure 6:**
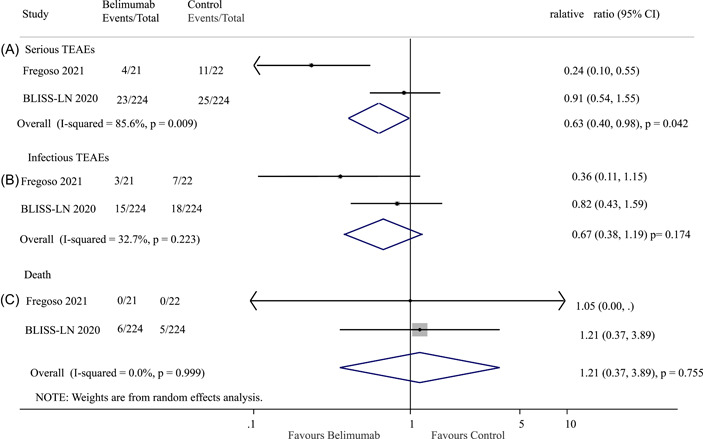
Effect of belimumab on (A) serious treatment‐emergent adverse events (TEAEs), (B) infectious TEAEs, and (C) death in patients with lupus nephritis. BLISS‐LN, Belimumab International Study in Lupus Nephritis; CI, confidence interval.

Two studies included in the analysis reported serious TEAEs.^21^, ^22^ Among the patients in the belimumab group, there were 27 (11.0%) serious TEAEs out of 245 patients, while in the control group, there were 36 (14.6%) serious TEAEs out of 246 patients. The results showed that belimumab treatment was associated with a 27% reduced risk of serious TEAEs compared to the control group (OR: 0.63; 95% CI: 0.40–0.98; *p* = 0.042; *I* ^2^ = 85.6%, *p* = 0.009; Figure 6A).

Meanwhile, the results indicated that belimumab therapy was not associated with a significant difference in infectious TEAEs compared to the control group (OR: 0.67; 95% CI: 0.38–1.19; *p* = 0.174; *I* ^2^ = 32.7%, *p* = 0.223; Figure 6B).

Overall, two trials provided data on death.^21^, ^22^ A total of six (2.4%) deaths occurred in the belimumab group out of 245 patients, while five deaths (2.0%) occurred in the active control group out of 246 patients. There was no statistical difference between belimumab and the control group in terms of mortality (OR: 1.21; 95% CI: 0.37–3.89; *p* = 0.775; *I* ^2^ = 0.0%, *p* = 0.999; Figure 6C).

### 3.8 Publication bias

The Begg funnel plot and Egger test indicated no evidence of publication bias for the outcome of renal improvement (*p* = 0.602; Supporting Information: Figure 2).

### 3.9 Case–control study

The baseline characteristics between the belimumab group and the standard therapy group were similar, as indicated in Table 3. There were no significant differences in age, gender, systemic pressure, baseline proteinuria, serum creatinine, and eGFR between the two groups. Additionally, the levels of active biomarkers, including anti‐dsDNA antibody, anti‐C1q antibody, C3, and C4, as well as the kidney biopsy class were comparable between the belimumab group and the standard therapy group. As for treatments, all patients in the two groups received standard therapy, including glucocorticoid and immunosuppressive therapy (Table 3).

**Table 3:** Baseline patients' characteristics in the case–control study.

Variable	Belimumab therapy (*n* = 6)	Standard therapy (*n* = 6)	*p* Value
Baseline characteristics			
Age (mean ± SD, year)	36.33 ± 5.50	38.50 ± 9.89	0.65
Gender (% female)	6 (100)	6 (100)	1
Systemic pressure (mmHg)	125 ± 9	126 ± 12	0.98
Proteinuria (mg/day)	3241 (2474, 7613)	2836 (700, 7451)	0.43
Serum creatinine (μmol/L)	128 ± 156.53	61.5 ± 35.42	0.33
eGFR (mL/min/1.73 m^2^)	101.67 ± 62.65	129.89 ± 51.00	0.41
Biomarkers			
Antidouble‐stranded DNA antibodies (IU/mL)	223.32 ± 110.79	106.98 ± 126.17	0.12
Anti‐C1q antibodies (U/mL)	42.28 ± 32.97	13.23 ± 11.53	0.26
Complement C3 (mg/dL)	29.52 ± 5.16	34.25 ± 10.78	0.35
Complement C4 (mg/dL)	4.97 ± 2.88	6.78 ± 4.84	0.45
Kidney biopsy class			
Class III or IV	4 (66.7)	4 (66.7)	1
Class III or IV and V	2 (33.3)	2 (33.3)	1
Treatment			
Belimumab dosage (mg)	8700 ± 1125		
Glucocorticoid (% yes)	6 (100)	6 (100)	1
Immunosuppressive therapy (% yes)	6 (100)	6 (100)	1

Abbreviation: eGFR, estimated glomerular filtration rate.

In the belimumab group, the median proteinuria levels showed a noticeable decrease from 3241 mg/day at baseline to 426 mg/day at Week 48 (*p* = 0.037), further decreasing to 299 mg/day at Week 72 (*p* = 0.016), and reaching 238 mg/day at Week 96 (*p* = 0.037). Conversely, in the control group, the median proteinuria levels decreased from 2836 mg/day at baseline to 1503 mg/day at Week 48, then to 1428 mg/day at Week 72, and finally to 1006 mg/day at Week 96 (Table 4). Treatment with belimumab was found to be associated with a stabilization of renal function (Table 4). Additionally, there was an increase in C3 and C4 levels over the course of 96 weeks in the belimumab group, suggesting a positive impact on the immune response and complement system. Furthermore, the use of belimumab resulted in a reduction in the dose of glucocorticoids at Weeks 24 and 48, indicating a potential steroid‐sparing effect.

**Table 4:** Summary of efficacy end points from baseline to 96 weeks treatment period in the case–control study.

Period (weeks)	Belimumab therapy	Standard therapy	*p* Value
Proteinuria (mg/day, median and IQR)			
12	2544 (1368.25, 7975.5)	4468 (637.75, 7604.25)	0.87
24	1645 (657, 5394)	3434 (1818, 6550)	0.63
48	426 (303.75, 1335.52)	1503 (1104, 2275)	0.037
72	299 (232.50, 567.62)	1428 (656.63, 2478)	0.016
96	238 (217.50, 390.25)	1006 (322.5, 1472.25)	0.037
eGFR (mL/min/1.73 m^2^, mean ± SD)			
12	112.81 ± 30.43	110.10 ± 27.82	0.88
24	121.27 ± 21.95	114.50 ± 12.69	0.53
48	118.93 ± 19.48	119.27 ± 10.42	0.97
72	123.36 ± 24.78	115.45 ± 10.50	0.49
96	113.04 ± 12.84	117.92 ± 8.53	0.46
C3 (mg/dL, mean ± SD)			
12	59.73 ± 14.50	82.48 ± 32.51	0.16
24	72.92 ± 4.86	74.24 ± 42.24	0.94
48	72.33 ± 7.49	81.65 ± 12.36	0.15
72	76.60 ± 10.99	85.87 ± 21.45	0.44
96	82.73 ± 14.08	80.15 ± 20.05	0.8
C4 (mg/dL, mean ± SD)			
12	12.93 ± 2.64	18.74 ± 9.34	0.19
24	16.92 ± 3.28	16.89 ± 9.70	0.99
48	19.47 ± 3.70	21.3 ± 5.62	0.52
72	19.92 ± 5.4	18.93 ± 1.50	0.77
96	19.73 ± 6.04	18.05 ± 10.34	0.74
Average daily prednisone‐equivalent dose (mg/day, mean ± SD)			
12	28.33 ± 6.83	34.16 ± 4.91	0.12
24	17.5 ± 2.23	21.67 ± 2.58	0.02
48	7.08 ± 2.45	12.91 ± 4.58	0.02
72	4.58 ± 1.02	6.25 ± 2.09	0.11
96	3.75 ± 1.37	4.17 ± 1.29	0.59

Abbreviations: eGFR, estimated glomerular filtration rate; IQR, interquartile range.

It is noteworthy that no severe adverse events were observed in either the belimumab group or the control group. This suggests that belimumab was well‐tolerated and did not lead to any significant safety concerns during the study period.

## 4 DISCUSSION

The therapeutic research on LN is indeed progressing toward precision medicine, with a focus on the evaluation of targeted biological drugs that aim to modulate specific pathways involved in the disease process.^23^, ^24^ In the context of this meta‐analysis, which included three trials and a total of 666 participants, our findings provide evidence that belimumab is associated with an increased likelihood of renal response and improvement in proteinuria. Furthermore, our analysis revealed that belimumab treatment led to a higher rate of positive‐to‐negative conversion for anti‐dsDNA antibodies, as well as normalization of low C3 and C4 levels, when compared to the control group. Importantly, treatment with belimumab was associated with a reduced risk of severe TEAEs. By including real‐life case–control studies in our analysis, we have enhanced the awareness of the beneficial renal response and the reduced occurrence of severe adverse events with the addition of belimumab to standard therapy in patients with LN. These findings provide valuable insights for clinicians and contribute to the growing body of evidence supporting the use of belimumab as a targeted therapy in the management of LN.

Among patients with SLE, the kidneys are frequently affected, and LN represents a significant challenge in their management. The primary goal of LN treatment is to achieve remission or, at the very least, minimize disease activity and preserve kidney function. However, the proportion of patients who achieve a satisfactory renal response remains unsatisfactory under current standard therapeutic approaches. The results of the BLISS‐LN (Belimumab International Study in Lupus Nephritis) trial have had a significant impact on the management of LN. These results led to the approval of belimumab, the first biologic agent, by both the Food and Drug Administration and European Medicines Agency) for the treatment of adult patients with active LN.^22^ The efficacy of belimumab in LN has been proven across several RCTs and previous meta‐analyses.^25^, ^26^ In the present study, the belimumab group increased the likelihood of renal response, renal complete response, and improvement in proteinuria. The biological significance of belimumab was also evident from the observed serological improvements. Recently, Shrestha et al.^26^ conducted a meta‐analysis to evaluate the efficacy and safety of belimumab in patients with LN. There are several notable differences between the present study and Shrestha's meta‐analysis. Shrestha's study included two trials conducted by Atisha‐Fregoso et al.^21^ and Furie et al.,^22^ whereas the present study included three trials. Additionally, Shrestha's study focused solely on renal response, while the present study expanded the evaluation to include not only renal response but also the conversion of active biomarker levels. By incorporating additional trials and expanding the outcome measures, the present study offers a more comprehensive analysis and a broader understanding of the effectiveness of the treatment intervention.

In addition to the meta‐analysis, we conducted a retrospective case–control study to further investigate the efficacy of belimumab in Chinese patients with LN. Our study showed that both the belimumab group and the control group experienced reductions in median proteinuria levels over time. However, the addition of belimumab to standard therapy resulted in significant improvements in kidney responses compared to standard therapy alone. It appears that the impact of belimumab in improving kidney responses took some time to become evident in our study. Specifically, the significant difference between the belimumab group and the control group was not observed until 48 weeks of treatment. The slow timing of the observed effects of belimumab in LN may be due to its mechanism of action. Belimumab targets and inhibits BAFF to affect B‐cell survival and proliferation. Since belimumab does not directly influence B cells, it may take some time for the drug to impact the B cell population and the overall immune response. Rituximab (RTX) is a monoclonal antibody that targets CD20‐expressing B cells and leads to their depletion. In one study, LN patients received sequential treatment with RTX and belimumab. This is based on the assumption that BAFF levels increase following RTX administration as a feedback mechanism; consequently, a sequential therapy should lead to the depletion of CD20‐expressing B cells and then prevent the rebounded B‐cell‐enhanced repopulation.^12^ The combination of the two medications may also have a synergistic effect. Belimumab is an effective medication for the prevention of LN.

The safety of belimumab therapy in patients with LN is a major concern.^27^ No safety signals were observed with regard to initiating or continuing belimumab treatment in patients with SLE or LN.^26^, ^28^ In this study, we found that treatment with belimumab was associated with a 27% reduction in the risk of serious TEAEs compared to placebo. In the belimumab group, the incidences of infectious TEAEs and death were not increased. No severe adverse events were observed in the belimumab group and the control group in the meta‐analysis, as well as in our LN patients. The reduction in the proportion of serious TEAEs observed with belimumab may be attributed to the lower daily glucocorticoid dose used. Treatment with glucocorticoid is associated with numerous potential side effects, such as infections, osteoporosis, and specific permanent organ damage. Glucocorticoids should be tapered quickly or discontinued whenever possible to minimize damage accrual while targeting renal remission or low disease activity and relapse.^29^ It is noteworthy that in our study, all patients received oral full doses of glucocorticoids at enrollment, and the addition of belimumab allowed for the tapering of glucocorticoid use in the early stages of the disease. By incorporating belimumab into the treatment regimen, it appears that patients were able to achieve better disease control and subsequently reduce their daily glucocorticoid doses after 24 weeks of treatment. Glucocorticoid use is a subjective aspect of LN treatment, influenced by factors such as physician experience and the availability of alternative effective therapies. The efficacy of belimumab plays a crucial role in guiding physicians toward successful glucocorticoid‐sparing strategies.

There are several limitations that should be considered when interpreting our results. First, it is regrettable that we were only able to include three RCTs in our analysis. We did not consider cohort or observational studies that may have reported outcomes with insufficient evidence. This limited pool of studies may affect the overall robustness and generalizability of our findings. Second, the small sample sizes in both groups pose a limitation. Small sample sizes can limit the statistical power of the study, making it more challenging to detect smaller effects or differences between the groups. Third, the lack of sufficient data on glucocorticoid doses in RCTs hampered the chance to evaluate the efficacy of belimumab in dose reduction or optimizing glucocorticoid therapy. Further research should strive to address these limitations to provide more accurate and reliable evidence regarding the efficacy and safety of belimumab in LN and its potential impact on glucocorticoid use.

## 5 CONCLUSION

In patients with LN, the utilization of belimumab therapy has demonstrated notable improvements in renal response rates. Additionally, it has shown a decreased likelihood of serious treatment‐related adverse events and a diminished need for glucocorticoid dosage when compared to the active control group. In addition to conventional immunosuppression, belimumab targeting the BAFF pathway offers a novel treatment for LN.

## AUTHOR CONTRIBUTIONS

Youxia Liu designed the study. Kai Zhang and Tiening Qi searched the paper. Kai Zhang, Tiening Qi, and Donghua Guo collected the data. Kai Zhang and Tiening Qi analyzed the results. Kai Zhang and Donghua Guo drafted the manuscript.

## CONFLICT OF INTEREST STATEMENT

The authors declare no conflict of interest.

## ETHICS STATEMENT

All subjects provided written informed consents. The study protocol was in adherence with the Declaration of Helsinki and was approved by the Institutional Ethical Committee of Tianjin Medical University General Hospital.

## Supporting information

Supporting information.

Supporting information.

## Data Availability

Raw data used during the current study are available from the corresponding author on reasonable request for noncommercial use.
