# Supplementary figures and images for: Efficacy and safety of belimumab therapy for patients with lupus nephritis: A meta‐analysis and a propensity score‐matched case–control study
Source: Immun Inflamm Dis. 2023 Jul 27;11(7):e954. doi: 10.1002/iid3.954 (PMC10373564; doi:10.1002/iid3.954)

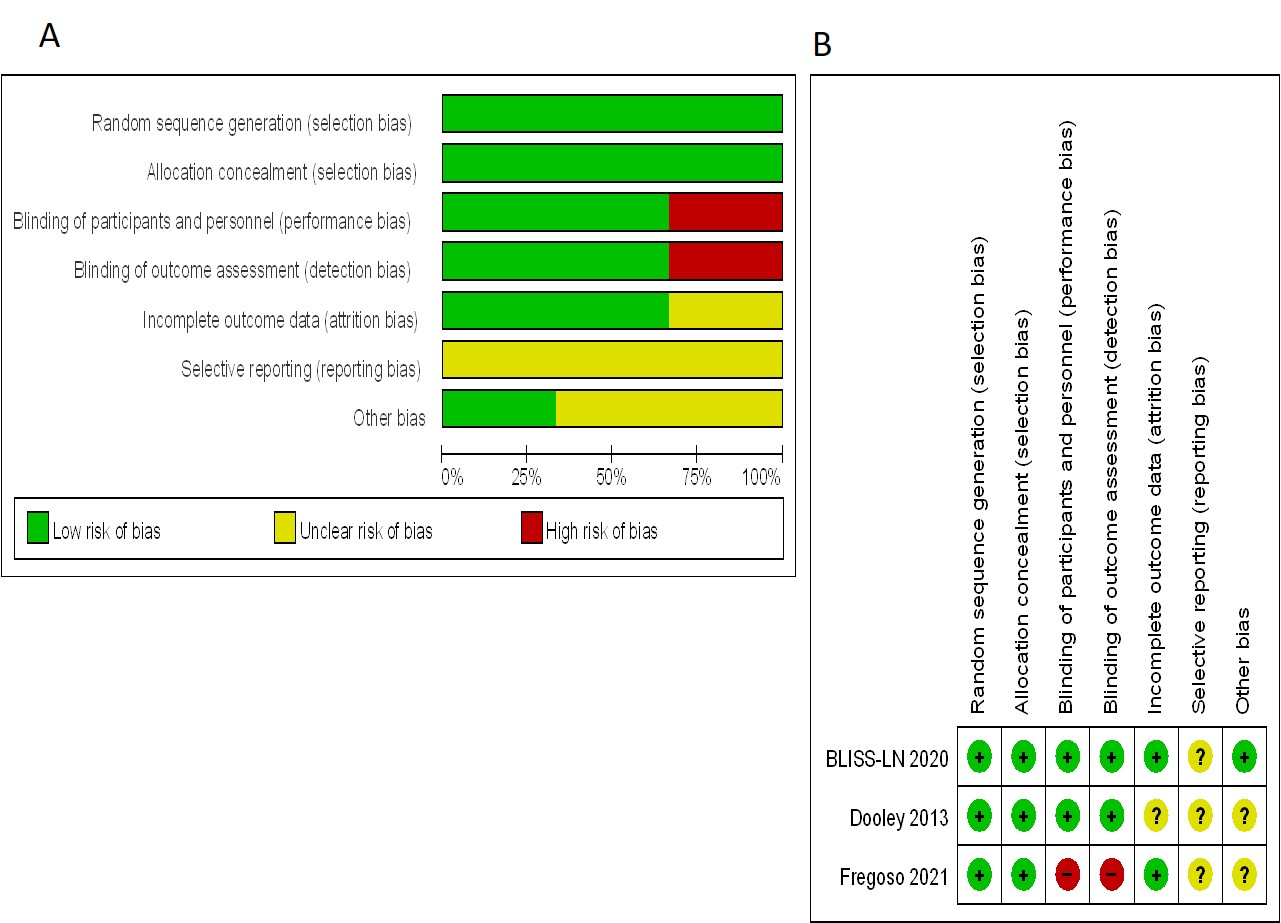

Supplement: Supplementary file 1 — Supporting information. [file IID3-11-e954-s001.jpg]

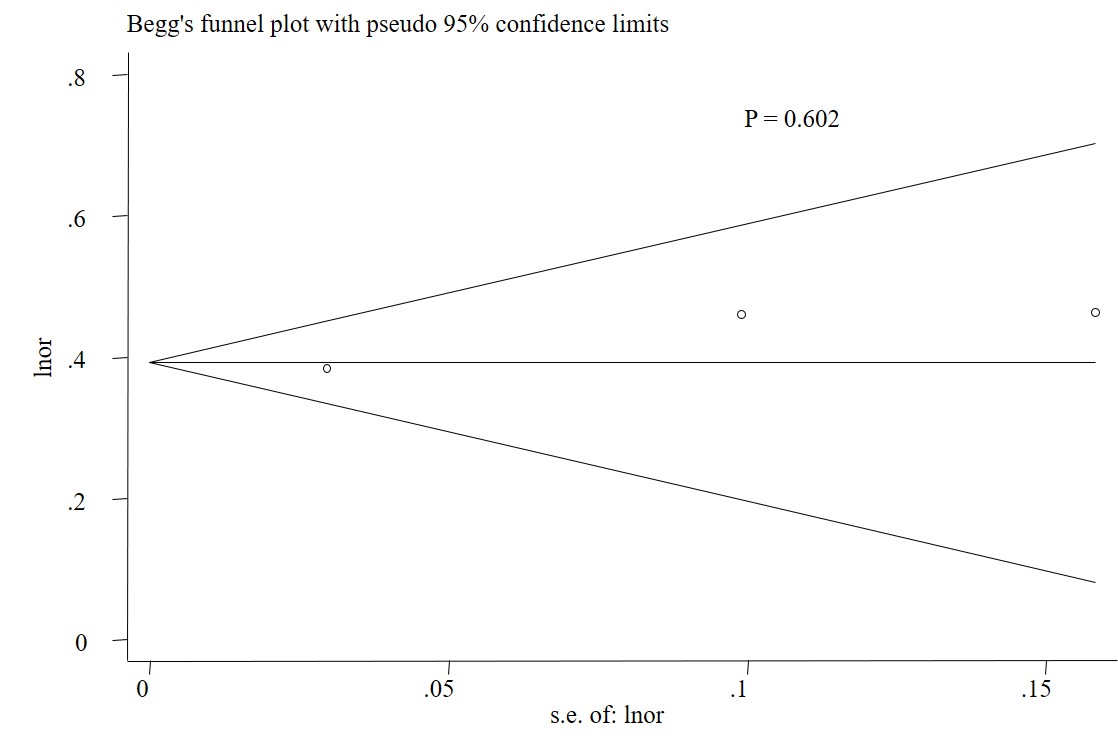

Supplement: Supplementary file 2 — Supporting information. [file IID3-11-e954-s002.jpg]
